# Lifestyle behavior clusters and their associations with depressive symptoms among Chinese adolescents: during and after COVID-19 period

**DOI:** 10.1080/21642850.2026.2677983

**Published:** 2026-06-01

**Authors:** Zeng-Bao Hu, Ke-Qin Ding, Stuart McDonald, Qing-Hai Gong, Yi Lin

**Affiliations:** a Faculty of Humanities and Social Sciences, University of Nottingham Ningbo China, Ningbo City, Zhejiang Province, China; b Department of School Health, Ningbo Municipal Center for Disease Control and Prevention, Ningbo City, Zhejiang Province, China

**Keywords:** Lifestyle behavior, adolescents, depressive symptoms, zero-COVID policy, China

## Abstract

**Background:**

The occurrence of depressive symptoms among adolescents presents a significant public health challenge. However, little is known about the influence of lifestyle changes on depressive symptoms among adolescents after the cancellation of the zero-COVID policy in China. This study aims to examine the associations between clusters of lifestyle behaviors and depressive symptoms among Chinese adolescents during and after the zero-COVID policy period.

**Methods:**

A school-based 2-year longitudinal study was conducted from September 2022 to October 2023 in Ningbo, Zhejiang Province, China. Depressive symptoms were assessed using the 20-item Center for Epidemiological Studies Depression Scale. Two-step cluster analysis was used to identify clusters based on adolescents' levels of moderate-to-vigorous physical activities, screen-based sedentary time, sleep duration, and consumption of sugar-sweetened beverages and deep-fried food in each period. A binary generalized linear model (GLM) regression was used to estimate and examine associations between lifestyle clusters and depressive symptoms.

**Results:**

A total of 2705 adolescents aged between 10 and 19 years were included in the analysis. The prevalence of depressive symptoms in the zero-COVID policy period was lower than that in the post-zero-COVID policy period (20.00% vs. 25.21%, *p* < 0.01). Five lifestyle clusters were recognized in each time period.

**Conclusions:**

In general, unhealthy lifestyle clusters were associated with a higher likelihood of depressive symptoms, with the association being stronger and more significant in the post-zero-COVID policy period. Effective interventions for adolescents' lifestyle behaviors, such as health education programs, are needed to reduce long-term negative effects on their mental health in the post-pandemic period and potential future pandemics.

## 1. Introduction

The occurrence of depressive symptoms among adolescents is a major global mental health challenge (World Health Organization [WHO], 2017). Adolescence (aged 10–19) is a unique and formative period for significant physiological, psychosocial, and cognitive changes, making adolescents vulnerable to mental problems such as depressive symptoms (Shorey et al., 2020; World Health Organization [WHO], 2024). Recent studies have indicated that more than one-fifth of children and adolescents globally had experienced depressive symptoms (Lu et al., 2024). In China, according to the National Mental Health Development (2021–2022), 14.8% of adolescents experienced depressive symptoms (Fu et al., 2023). There is also evidence that the COVID-19 pandemic has had a profound negative impact on adolescents' mental health, with studies reporting increases in the prevalence of depressive symptoms among adolescents during the pandemic period in many regions, such as the U.S.A. (Hawes et al., 2022), Europe (Ludwig-Walz et al., 2022), Canada (Gohari et al., 2024), and China (Du et al., 2024). Studies based on data collected before and during the pandemic have suggested that unhealthy lifestyle behaviors are important risk factors for depressive symptoms (Bui et al., 2023, 2024; Cao et al., 2020). In particular, insufficient sleep duration (SLD), prolonged screen-based sedentary time (SST), reduced moderate-to-vigorous physical activity (MVPA), consumption of sugar-sweetened beverages (SSB), and deep-fried food (DFF) have been identified as major risk factors associated with a higher risk of depressive symptoms either independently or jointly (Jin et al., 2024; Lee et al., 2024; Liao et al., 2021; Lu et al., 2020; Xiang et al., 2022).

During the COVID-19 period, over 1.6 billion adolescents in more than 190 countries were affected by school closures caused by emergent containment measures implemented by governments (UNSDG, 2020). Especially, China implemented the strict zero COVID-19 policy from February 2020 to December 2022 (Gong et al., 2024). Under the zero COVID-19 policy, comprehensive emergency control measures, including nationwide nucleic acid testing for adults and school-aged children, school closure, and extended periods of quarantine imposed on local residents, were implemented in response to infection cases being detected in local populations so as to prevent the spread of the virus (Ding & Zhang, 2022). It was estimated that over 220 million Chinese children and adolescents were affected by these measures and were forced to stay at home for online education (Wang et al., 2020), which dramatically changed the lifestyle behaviors of adolescents during the pandemic period (Scapaticci et al., 2022). For example, some adolescents were quarantined at home, leading to self-reported increases in electric device use and a decrease in physical exercise (Guo et al., 2024). The dietary patterns of adolescents were also changed during home quarantine (Yu et al., 2021). Although these control measures were discontinued in China after the cancellation of the zero COVID-19 policy in December 2022, it has been argued that the negative impacts on adolescents' lifestyles and mental health may persist long in the post-COVID-19 period (Melchior, 2023). Worldwide, studies have also found that homeschooling during the COVID-19 pandemic has changed adolescents' lifestyle behaviors and caused relevant psychological and emotional problems (Deng et al., 2023; Panda et al., 2021).

However, most studies, internationally and in China, have focused on the changing lifestyles and depressive symptoms of adolescents during the COVID-19 period (Jin et al., 2024; Lee et al., 2024; Liao et al., 2021; Lu et al., 2020; Xiang et al., 2022), and few comparative studies have focused on the pandemic and post-pandemic periods. These two periods differ significantly in terms of social context: the zero-COVID period was characterized by strict government-mandated restrictions, such as school closures and home quarantine, which affected many adolescents; the post-zero-COVID period was characterized by the absence of these restrictions. The policy shift provides a unique opportunity to examine how changes in public health policies and related social contexts affect the relationship between individual lifestyle behaviors and mental health. The socio-ecological model (SEM) suggests that individual lifestyle behaviors are not simply personal choices but are influenced by interacting multi-level environments, including family, school, and government regulations (Bronfenbrenner, 1975; Stokols, 1992). During the zero-COVID period, students experienced home quarantine and homeschooling as part of pandemic control measures, creating a significantly different environment than the post-zero-COVID period and, as a result, different lifestyle behavior patterns among adolescents. Specifically, research has shown that restrictions during the pandemic had substantial impacts on adolescents' lifestyle behaviors, such as less physical activity, more electric device usage, and unhealthy dietary patterns (Guo et al., 2024; Yu et al., 2021). Given the important impact of lifestyle on mental health among adolescents (Jin et al., 2024; Lee et al., 2024; Liao et al., 2021; Lu et al., 2020; Xiang et al., 2022), a comprehensive understanding of the relationship between changing lifestyle behaviors and mental health during and after the pandemic period is necessary to assist the government, schools, and health professionals in developing targeted policies and intervention measures to improve adolescents' well-being in the post-pandemic period and to prepare for potential future pandemics. However, it remains unclear whether the associations between lifestyle behaviors and depressive symptoms differ between these periods.

To fill this research gap, the present study aims to examine and compare the clusters of MVPA frequency, SST, SLD, and consumption of SSB and DFF in Chinese adolescents before and after the cancellation of the zero COVID-19 policy and their associations with depressive symptoms. Our research questions are ‘How are the patterns of lifestyle behaviors among adolescents associated with depressive symptoms among adolescents during the zero-COVID and post-zero-COVID periods?’ and ‘Do these associations differ between the two periods?’. Based on the above discussions on adolescents' lifestyle, mental health, and changing environment due to policy shifts, we propose the following hypotheses:

H1: Unhealthy lifestyle clusters are associated with a higher likelihood of depressive symptoms in the zero-COVID policy period.

H2: Unhealthy lifestyle clusters are associated with a higher likelihood of depressive symptoms in the post-zero-COVID policy period.

H3: The association between unhealthy lifestyle clusters and depressive symptoms was stronger in the post-zero-COVID policy period.

## 2. Materials and methods

### 2.1. Study design and participants

**Figure 1.**
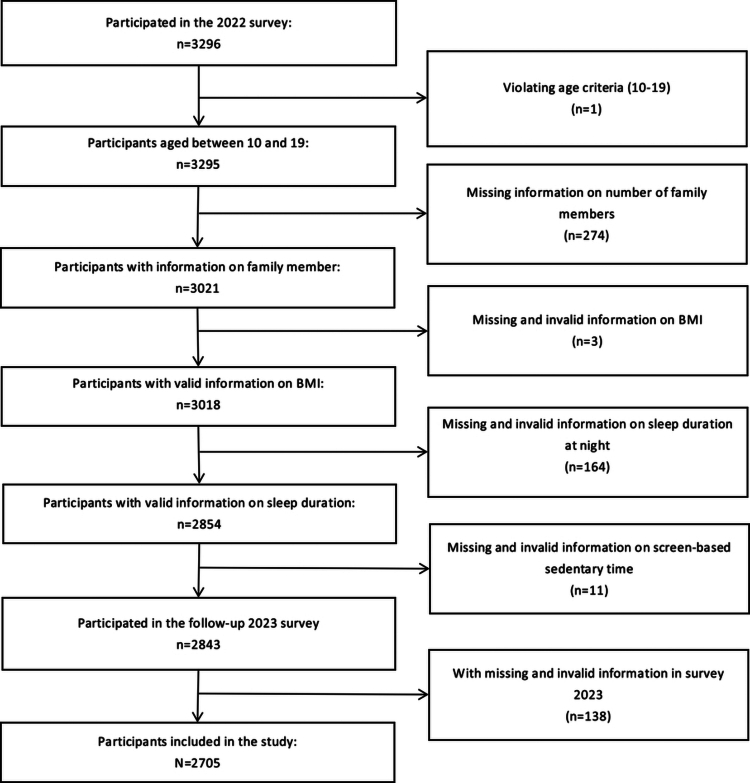
Flow chart of inclusion of sample in this study.​

Utilizing a school-based 2-year longitudinal study conducted in Ningbo, China, among adolescents from September 2022 to October 2023, the paper constructs a two-period longitudinal dataset. A multistage, stratified cluster sampling procedure was utilized to draw the target samples, covering socio-economic status (SES), demographic, health, and psychological aspects. Two junior middle schools, two senior middle schools, and one vocational high school were randomly selected from each district, while two middle schools and one senior middle school were randomly selected from each county. Two classes per grade were randomly selected from each school. A total of 11,853 adolescents participated in the baseline study from September to October 2022 (Wave 1), while 11976 participated in the study from September to October 2023 (Wave 2) (Lin et al., 2025). For the purpose of our research, 3296 students who participated in both waves were included in the present study. As shown in Figure 1, after applying the exclusion criteria and removing cases with missing or invalid data, 2705 students who participated in both Wave 1 and Wave 2 were included in the present study, with a dropout rate of 17.93%.

The inclusion criteria for adolescents (see Figure 1) were: (1) students aged 10–19 years; (2) being able to understand the questions in the questionnaire and complete the questionnaire independently; and (3) the written informed consent from both the student and a parent or legal guardian of the student for their participation in this study. The exclusion criteria were as follows: (1) students who participated in the survey without consent and (2) students who were sick or had injuries affecting the health examination. At the baseline study and follow-up study, all the students attended health examination conducted by trained medical professionals in the early morning at the schools. A comprehensive set of objective health indicators, including height, weight, blood pressure status, visual acuity, oral health, spinal health, and lung volume, was collected.

Students were measured in light clothing and barefoot by medical professionals. Body weight was measured using an electronic scale to the nearest 0.1 kg, and height was measured using a free-standing stadiometer to the nearest 0.1  cm. After the health examination, all the students participated in the Wave 1 and Wave 2 surveys. Researchers were trained before they administered the survey. Before the students filled out the self-administered anonymous questionnaires, henceforth referred to as questionnaires, an introduction and the instructions for the surveys were provided to all the students from the selected classrooms by experienced researchers. During the Wave 1 and Wave 2 surveys, all the students were asked to complete the questionnaires independently within 40 minutes in their classrooms under the supervision of public health specialists, who were available to provide standardized clarifications if any participant had questions about the questions. All submitted information was double-checked for quality control by the specialists. Missing or misreported information was re-requested during the survey. To enable data linkage across two waves while preserving anonymity, each participant was assigned a unique study-specific identification number at both waves.

### 2.2. Ethics statement

Ethical approval for this study was approved by the ethical committee for this research (No. 202201) and followed the Declaration of Helsinki. Written or verbal informed consent was obtained from all the students, their parents, or legal guardians. All the information related to the students was confidential.

### 2.3. Measures

#### 2.3.1. Depressive symptoms

Depressive symptoms were assessed by using the Chinese version of the Centre for Epidemiological Studies-Depression (CES-D) scale, a 20-item questionnaire that assesses the status of depressive symptoms for the past seven days. The CES-D was previously validated and has been widely used with good reliability and validity in Chinese students (Guo et al., 2014; William Li et al., 2010). Students were asked to report the frequency with which each depressive symptom was experienced during the past week (e.g. restless sleep, poor appetite, and mood that they experienced over the previous week). The CES-D has 4 response options with scores ranging from 0 to 3 for each item: 0-rarely or none of the time (<1 day), 1-some or little of the time (1–2 days), 2-moderately or much of the time (3–4 days), and 3-most or almost all the time (5–7 days). The range of the total score is from 0 to 60, with high scores indicating greater depressive symptoms (Radloff, 1977) and the cut-off value of 16 identifying individuals with depressive symptoms (Lewinsohn et al., 1997).

#### 2.3.2. Physical activity

The level of physical activity was assessed by the following question: ‘How many days were you able to perform at least 60 minutes of MVPA (moderate-to-vigorous physical activities) over the past week?’. To ensure that adolescents understood the question, it was accompanied by a plain-language explanation and age-appropriate examples: ‘Moderate-to-vigorous physical activities refer to activities that make you breathe harder or make your heart beat faster, such as running, basketball, football, swimming, aerobics, or lifting heavy objects.’ MVPA was categorized into two groups: 1) <7 days/week, 2) 7 days/week (Chaput et al., 2020).

#### 2.3.3. Screen-based sedentary time

SST (screen-based sedentary time) was assessed by the question ‘How long did you use electronic devices over the past week, including watching television, using computers, and playing video games?’. According to the Canadian 24-hour movement guidelines, the time spent on electronic devices meeting the SST guideline requires less than 2 hours per day (Tremblay et al., 2016). SST was categorized as: 1) <2 hours/day as short SST, 2) ≥2 hours/day as excessive SST.

#### 2.3.4. Sleep duration

All the students reported their average sleep duration (SLD) every night for the past week. SLD was initially categorized into four groups based on the recommendation proposed by the National Sleep Foundation: 1) very short SLD (SLD < 7 and <6 hours per night for students aged ≤13 years and students aged 14–19 years, respectively); 2) short SLD (SLD of 7–8 and 6–7 hours per night for students aged ≤13 and students aged 14–19 years, respectively); 3) recommended SLD (SLD of 9–11 and 8–10 hours per night for students aged ≤13 years and students aged 14–19 years, respectively); and 4) long SLD (SLD > 11 and >10 hours per night for students aged ≤13 years and students aged 14–19 years, respectively) (Chaput et al., 2018; Hirshkowitz et al., 2015). The majority of adolescents (approximately 93%) reported sleep durations between 6 and 9 hours, and the number of cases in extreme categories, such as ‘very short’ and ‘long’, was too small. Therefore, SLD was categorized into two groups based on wehter adolescents met the recommended sleep duration: (1) insufficient SLD, comprising those who failed to meet the age-specific recommendation sleep duration (i.e. the ‘very short’ and ‘short’ groups); (2) sufficient SLD, comprising those who met the age-specific recommendation sleep duration (i.e. the ‘recommended’ and ‘long’ groups).

#### 2.3.5. Sugar-sweetened beverage consumption

The consumption of SSB (sugar-sweetened beverage) was evaluated by the question ‘How often did you drink soda and other sugar-added beverages for the past week, including Coca Cola, Iced Black Tea, Minute Maid over the past week?’ Based on the evidence documenting the significant mental health risks associated with daily consumption of SSB (Freije et al., 2021; Urugo et al., 2026), SSB was categorized as: (1) 0 or <1 time/day as low SSB; (2) ≥1 time/day as high SSB.

#### 2.3.6. Deep-fried food consumption

The question ‘How often did you have deep-fried foods for the past week’ was used to assess the frequency of deep-fried food (DFF) consumption. Based on previous studies on the significant association between daily DFF consumption and mental health problems (Ejtahed et al., 2024), DFF was categorized as: (1) 0 or <1 time/day as low DFF; and (2) ≥1 time/day as low DFF.

### 2.4. Covariates

Body mass index (BMI) was calculated with the following formula: weight (kg)/height^2^ (m^2^). The BMI z-score was calculated to standardize the BMI value across sex and age groups (World Health Organization [WHO], 2007). Students were asked to report information on demographics [sex, age, school type (junior middle school, senior middle school/vocational high school)], family structure (number of family members living together), and breakfast habits (whether regularly eating breakfast). Blood pressure status was measured by medical professionals during the health examination conducted on the same day prior to the survey.

### 2.5. Statistical analysis

The data in the present study are presented as numbers and percentage form for categorical variables and means with standard deviation (SD) for continuous variables. The subgroup differences in the percentage and prevalence were tested using the Pearson chi-square (*Χ*²) test. Student's *t*-test was used to compare means between different subgroups.

For sensitivity analysis, Cronbach's alpha was used to test the validation of CES-D questions used for Chinese adolescents, and the Cronbach's alpha in this study was 0.85, identifying the internal reliability of CES-D questions. Prior to cluster analysis, all lifestyle behavior variables were transformed into binary indicators (healthy vs. unhealthy) based on public health guidelines and previous literature, as detailed in Section 2.2. This transformation could place them on the same scale, thus eliminating the concerns for comparability. A two-step cluster analysis was used to categorize the samples into different classes based on the lifestyle behaviors. The optimal number of clusters can be determined by automatic analysis. Log-likelihood was used to measure cluster distance, and the clustering criterion of the Bayesian information criterion (BIC) was used to perform the clustering steps. In this study, the number of clusters was determined according to the best combination using BIC, BIC changes, high ratio of BIC changes, and a high ratio of distance measures after automatic clustering analysis. The silhouette coefficient was higher than 0.50, indicating a good model in this study.

The binary generalized linear model (GLM) was used to examine the associations between depressive symptoms and lifestyle behavior groups, adjusting for adolescents' demographic factors, family structure, BMI, blood pressure status and breakfast habits.

The results were considered statistically significant at a two-tailed level of 0.05. Statistical analysis was conducted using the STATA statistical software package V.17 (2021), and cluster analysis was performed using IBM SPSS Statistics V.28.

## 3. Results

### 3.1. Demographic characteristics of participants

Table 1 displays the demographic characteristics of the 2705 adolescents in the present study, of which 52.20% were boys. The average age of the adolescents in the zero-COVID policy period was 14.22; 67.84% of them were living with 3–5 family members, 57.07% were from middle school, 63.66% had a normal blood pressure status, and 67.62% had a normal weight.

**Table 1.** Demographic characteristics of the participants during and after the zero-COVID policy period.

	Zero-COVID policy period^a^	Post-Zero-COVID policy period^b^	*p*-value
*n* (%)			
Sex			–
Girls	1293 (47.80)	1293 (47.80)	
Boys	1412 (52.20)	1412 (52.20)	
Number of family members living together			0.718
<3	705 (26.06)	711 (26.28)	
3–5	1835 (67.84)	1843 (68.13)	
>5	165 (6.10)	151 (5.58)	
School type			0.483
Middle school	1543 (57.04)	1499 (55.42)	
High school	826 (30.54)	857 (31.68)	
Vocational high schools	336 (12.42)	379 (12.90)	
Blood pressure			0.021
Normal	1722 (63.66)	1803 (66.65)	
High	983 (36.34)	902 (33.35)	
Weight Status			0.420
Underweight	50 (1.85)	48 (1.77)	
Normal weight	1829 (67.62)	1874 (69.28)	
Overweight and obesity	826 (30.54)	783 (28.95)	
Sugar-sweetened beverage consumption (time/day)			–
No or <1	2483 (91.79)	2483 (91.79)	
≥1	222 (8.21)	222 (8.21)	
Physical activity (days/week)			0.105
<7	2414 (89.24)	2376 (87.84)	
7	291 (10.76)	329 (12.16)	
Screen-based sedentary time (hours/day)			0.423
<2	2150 (79.48)	2026 (78.60)	
≥2	555 (20.52)	579 (21.40)	
Sleep duration over night			0.891
Short	1150 (42.51)	1145 (42.33)	
Sufficient	1555 (57.49)	1560 (57.67)	
Consumption of fried fast food (time/day)			0.264
<1	2590 (95.75)	2606 (96.34)	
≥1	115 (4.25)	99 (3.66)	
Have breakfast everyday			0.115
Yes	2328 (86.06)	2287 (84.55)	
No	377 (13.94)	418 (15.45)	
Depressive symptoms			<0.001
No	2164 (80.00)	2023 (74.79)	
Yes	541 (20.00)	682 (25.21)	
Mean (SD)			
Age (years)	14.22 (1.56)	15.05 (1.55)	<0.001
BMI (kg/m^2^)	20.87 (3.91)	21.40 (4.00)	<0.001
CES-D	11.55 (6.69)	12.20 (8.13)	0.001

Note: Comparisons between groups were conducted using chi-square test for category variables, and using *t*-test for continuous variables.

^a^ Zero-covid period denotes the year 2022, when the zero-covid policy was in effect.

^b^ Post-zero-covid policy period denotes the year 2023, when the zero-covid policy was discontinued.

### 3.2. Characteristics of lifestyle behaviors and depressive symptoms

The lifestyle behaviors and depressive symptoms of the 2705 adolescents are shown in Table 1. During the zero-COVID policy period, 8.21% and 4.25% of the adolescents reported having SSB consumption and DFF intake at least once a day, respectively; 89.24% failed to engage in 60-minute MVPA every day, 20.52% reported an SST time of more than 2 hours a day, 42.51% had insufficient sleep over night, and 13.94% did not have breakfast every day. The changes in lifestyle behaviors of the adolescents were not significantly different between the zero-COVID policy period and after the zero-COVID policy period. However, the overall prevalence of depressive symptoms was reported to have significantly increased from 20.00% in the zero-COVID policy period to 25.21% in post-zero-COVID policy period (*p* < 0.001).

### 3.3. Clusters of lifestyle behaviors

The label of the lifestyle clusters was identified based on the most accentuated behaviors. The two-step cluster analysis suggested that a 5-class model had good cohesion and separation of clusters in both zero-COVID policy and post-zero-COVID policy periods, with silhouette coefficients greater than 50%. Lifestyle behavior in a cluster is considered as healthy if its probability is greater than 50%; therefore, a cluster is healthier if it has more lifestyle behaviors with probabilities higher than 50%.

**Figure 2.**
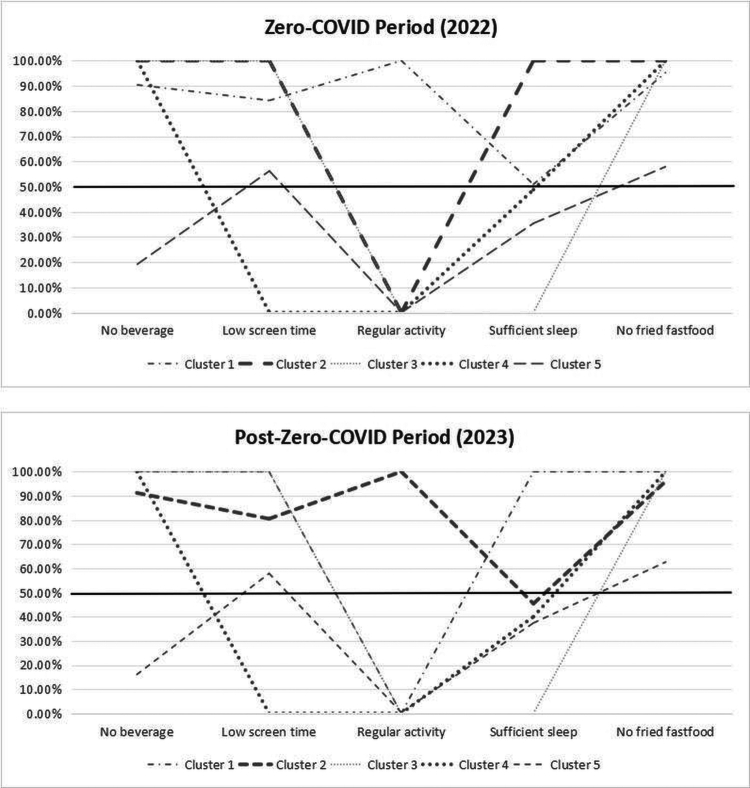
Classes of clustered lifestyle behaviors during and after the zero-COVID policy period.

Figure 2 displays the clusters of lifestyle behaviors in the two periods, respectively. Adolescents in Cluster 1 had the healthiest lifestyle during the zero-COVID policy period, with the probabilities of all lifestyle behaviors being greater than 50%. Cluster 2 was classified as the physically inactive group, Cluster 3 was the physically inactive and insufficient sleep group, Cluster 4 was physically inactive, having excessive SST, and having insufficient sleep, while Cluster 5 was characterized as the excessive SSB, physically inactive, and insufficient sleep group. In the post-zero-COVID policy period, Cluster 1 was the physically inactive group, Cluster 2 was classified into the insufficient sleep group, Cluster 3 was the physically inactive and insufficient sleep group, Cluster 4 was the excessive SST, physical inactivity, and insufficient sleep group, and Cluster 5 was the excessive SSB, physically inactive, and insufficient sleep group.

### 3.4. The prevalence of depressive symptoms across clusters

There were significant differences in the prevalence of depressive symptoms across clusters in both the zero-COVID policy period and the post-zero-COVID policy period (*p* < 0.001). The highest prevalence of depressive symptoms was observed in Cluster 5 in both periods (34.17% and 39.13%, respectively), followed by Cluster 4 (23.51% and 32.06%, respectively) and Cluster 3 (21.05% and 29.21%, respectively). The prevalence of depressive symptoms was higher in Cluster 1 (19.24%) than in Cluster 2 (12.08%) during the zero-COVID policy period. The prevalence rates were 22.26% and 13.06% in Cluster 2 and Cluster 1, respectively, in the post-zero-COVID policy period (Table 2).

**Table 2.** The prevalence of depressive symptoms among clusters during and after the zero-COVID policy period.

Zero-COVID policy period (2022)				
	Non-depressive symptoms (*n* = 2164)	Depressive symptoms (*n* = 541)	Prevalence of depressive symptoms	*p-*value^a^
Cluster 1	235 (10.86)	56 (10.35)	19.24%	<0.001
Cluster 2	633 (29.25)	87 (16.08)	12.08%	
Cluster 3	829 (38.31)	221 (40.85)	21.05%	
Cluster 4	309 (14.28)	95 (17.56)	23.51%	
Cluster 5	158 (7.30)	82 (15.16)	34.17%	

Post-zero-COVID policy period (2023)				
	Non-depressive symptoms (*n* = 2023)	Depressive symptoms (*n* = 682)	Prevalence of depressive symptoms	*p-*value^a^
Cluster 1	646 (31.93)	97 (14.22)	13.06%	<0.001
Cluster 2	255 (12.61)	73 (10.70)	22.26%	
Cluster 3	698 (34.50)	288 (42.23)	29.21%	
Cluster 4	284 (14.04)	134 (19.65)	32.06%	
Cluster 5	140 (6.92)	90 (13.20)	39.13%	

^a^ Comparisons were made between clusters using the chi-square test.

### 3.5. Associations between clustered lifestyle behaviors and depressive symptoms

According to the crude model in Table 3, in the zero-COVID policy period, only Cluster 2 and Cluster 5 were associated with the risk of depressive symptoms, while all cluster patterns were associated with the risk of depressive symptoms in the post-zero-COVID policy period. After controlling age, sex, school type, number of family members living together, BMI, blood pressure status, and breakfast habits, the associations of the same clusters maintained significant in the adjusted model in both periods. In the zero-COVID policy period, when compared with Cluster 1, Cluster 5 was associated with the higher odds of depressive symptoms (OR: 1.69, CI: 1.1–2.57), but Cluster 2 was associated with a lower risk of depressive symptoms (OR: 0.52, CI: 0.36, 0.75). In the post-zero-COVID policy period, Cluster 5 was associated with the highest odds of depressive symptoms (OR: 3.43, CI: 2.40–4.90), followed by Cluster 4 (OR: 2.72, CI: 1.98–3.73), Cluster 3 (OR: 2.22; CI: 1.71–2.88), and Cluster 2 (OR: 1.93, CI: 1.37–2.73).

**Table 3.** Associations between clusters of lifestyle behaviors and depressive symptoms during and after the zero-COVID policy period.

Clusters of lifestyle behaviors	Zero-COVID policy period (2022)				Post-zero-COVID policy period (2023)			
	Crude^a^		Adjusted^b^		Crude^a^		Adjusted^b^	
	OR	95% CI	OR	95% CI	OR	95% CI	OR	95% CI
Cluster 1 (ref)	1		1		1		1	
Cluster 2	0.58	0.40,0.83	0.52	0.36,0.75	1.91	1.36,2.67	1.93	1.37,2.73
Cluster 3	1.12	0.81,1.55	1.03	0.74,1.44	2.75	2.13,3.54	2.22	1.71,2.88
Cluster 4	1.29	0.89,1.87	1.06	0.71,1.56	3.14	2.34,4.23	2.72	1.98,3.73
Cluster 5	2.18	1.47,3.23	1.69	1.11,2.57	4.28	3.05,6.02	3.43	2.40,4.90

Note: OR: odds ratio; CI: confidence interval.

A binary GLM model was used to investigate the associations.

^a^ Crude: the crude model, without controlling for any covariate.

^b^ Adjusted: the model controlled for age, sex, school type, number of family members living together, BMI, blood pressure status, and breakfast habit.

## 4. Discussion

The present study investigated and compared the associations between lifestyle behaviors and depressive symptoms among Chinese adolescents during and after the zero COVID-19 policy period. Lifestyle behaviors were classified into five distinct clusters in each period, based on the levels of MVPA, SST, SLD, and consumption of SSB and DFF. Clusters were recognized from the healthiest (Cluster 1) to the unhealthy (Cluster 5) according to different combinations of lifestyle behaviors in each period. A GLM regression was then used to estimate the associations between lifestyle clusters and depressive symptoms. The study found that, in general, adolescents in unhealthy lifestyle clusters had a higher likelihood of suffering depressive symptoms. However, the associations exhibited different patterns in the two periods, with unhealthier clusters exhibiting stronger and more significant associations with the risk of depressive symptoms in the post-zero-COVID policy period.

The prevalence of depressive symptoms among Chinese adolescents in our study was 20.00% and 25.21% in the zero-COVID policy period and post-zero-COVID policy period, respectively. The prevalence in the zero-COVID policy period in our study was lower than those in studies conducted in Yunnan (36.33%) and Guangzhou (29.2%) of China, using the CES-D to measure depressive symptoms (Wang et al., 2022; Yang et al., 2024). A study among U.S.A. adolescents during the 2020 pandemic period, which used the CES-D method, reported a prevalence of 26.5%, which is higher than our result (Schwartz-Mette et al., 2023). This might be because our study was conducted at the later stage of the pandemic period (2022). The pandemic control policies were executed less strictly than those in the early stage, which mitigated the negative impacts on local adolescents' mental health.

One interesting finding of the present study is that the prevalence of depressive symptoms among adolescents increased significantly after the cancellation of zero-COVID policy. One possible explanation is that the persistent negative impact of COVID-19 has deteriorated adolescents' mental health during the post-pandemic period (Melchior, 2023). According to a recent study, many people who had COVID-19 experienced worse cognitive and psychiatric symptoms 2–3 years after infection, including depression, anxiety, and fatigue (Taquet et al., 2024). Besides, strict pandemic restrictions, such as lockdown policies, have been found to yield mental health problems, including an increase in depressive symptoms and anxiety (Keisari et al., 2022). Ningbo did not experience strict lockdowns during 2022, which may partly explain the lower prevalence of depressive symptoms of our sample during the zero-COVID policy period.

Our study did not find significant changes in adolescent lifestyle behaviors, including the frequency of engaging in physical activities and consuming SSB and DFF, SST, and SLD, between during and after the zero-COVID policy period. However, the combination of lifestyle behaviors of adolescents exhibited different patterns across the two periods. We identified five clusters of lifestyle behaviors in each period, ranging from the healthier cluster to the unhealthy cluster. In general, cluster 1 was identified as the healthy group in the zero-COVID policy period, while, no such healthy cluster was observed in the post-zero-COVID period. Studies in Hong Kong (Zhu et al., 2021), Italy (Di Renzo et al., 2020), and Scotland (Williams et al., 2021) observed positive lifestyle changes during the pandemic outbreak, including more exercise time, healthier eating behaviors, and more supportive interactions with family, which has supported our findings. However, the opposite results from some studies have shown that adolescents' lifestyle became significantly unhealthy during the pandemic period due to homeschooling (Guo et al., 2024; Jia et al., 2021; Scapaticci et al., 2022; Yu et al., 2021). This difference might be explained by the increased awareness of a healthy lifestyle of adolescents and their families during the pandemic in Ningbo.

Our results showed the co-existence of healthy and unhealthy lifestyle behaviors in the same cluster. For instance, adolescents in cluster 4 were identified as physically inactive, having excessive SST and insufficient sleep, but not consuming SSB and DFF, in both periods. This finding is supported by the theory of compensatory health beliefs (CHBs), where people believe that the negative health impacts of unhealthy behaviors could be offset by engaging in healthy behaviors (Knäuper et al., 2004). This belief may lead to a cycle of unhealthy behaviors followed by compensatory healthy behaviors, potentially hindering the development of healthy lifestyles. Another finding is the co-occurrence of related unhealthy lifestyle behaviors. For example, adolescents in cluster 4 in both periods were prone to have excessive SST and insufficient sleep simultaneously, and those in cluster 5 were found to be physically inactive and with excessive SSB consumption. The co-occurrence of unhealthy behaviors corresponds to the theories of triadical influence (TTI) and the transfer theory (TT) (Lippke et al., 2012). According to the TTI, some unhealthy behaviors are closely connected due to similar etiologies and health consequences and are therefore likely to occur together within a cluster. For instance, consumption of SSB could negatively affect physical fitness, making individuals less likely to engage in physical activities (Malik et al., 2013). Besides, the TT implies that individuals can change their behaviors only if the two domains share enough similarities, which explains why it is difficult for individuals with unhealthy behaviors to switch to healthier ones.

In general, adolescents of relatively healthier lifestyle clusters are less likely to suffer depressive symptoms in both zero-COVID policy and post-zero-COVID policy periods. This finding is consistent with a recent meta-analysis that revealed a significant association between unhealthy clusters of lifestyle behaviors and increased odds of depressive symptoms among adolescents (Efa et al., 2025). In particular, our results suggested that the combination of unhealthy lifestyle behaviors, such as lacking MVPA, insufficient SLD, excessive SST, and over-consumption of SSB, could increase adolescents' likelihood of having depressive symptoms. This finding is supported by previous studies that identified them as important risk factors for adolescents' depressive symptoms (Jin et al., 2024; Lee et al., 2024; Liao et al., 2021; Lu et al., 2020; Xiang et al., 2022). However, the associations between unhealthy clusters and the risk of depressive symptoms were stronger and more significant in the post-zero-COVID policy period. There are several explanations for this difference. First, studies found that people had more quality time to spend with family and more support from family members during the pandemic period (Di Renzo et al., 2020; Zhu et al., 2021). The positive changes in more interaction with family during the pandemic may help mitigate the negative impacts of an unhealthy lifestyle on depressive symptoms, as family support was found to play a critical role in preventing and alleviating depressive symptoms (Kamen et al., 2011). Second, unhealthy behaviors could be used to regulate emotions during the pandemic period, which may alleviate the association between unhealthy behaviors and depressive symptoms. A study of 5882 German children and adolescents suggested that SSB was used to deal with stress (Kadel et al., 2020). Besides, previous studies have suggested that positive smartphone content could provide temporary distraction to negative emotions (Rideout & Fox, 2018) and the entertainment use of smartphones could improve adolescents' mood in the short-term (Rodman et al., 2024). Particularly, a study of 14,789 Chinese college students during the pandemic period revealed a U-shaped relationship between SST and depressive symptoms, with SST potentially reducing depressive symptoms in the short run (Zhang et al., 2021).

It is noteworthy that adolescents of the ‘physically inactive’ cluster (Cluster 2) were less likely to experience depressive symptoms compared to those of the ‘healthy’ cluster (Cluster 1) during the zero-COVID policy period. Our results differ from previous studies, as previous studies have suggested that engaging in physical activities could reduce the likelihood of having depressive symptoms among adolescents (Recchia et al., 2023). This counter-intuitive finding might be explained by the disruption of regular exercises. Adolescents of the ‘healthy’ cluster may face greater mental distress due to the disruption of their regular exercise routines caused by the pandemic control measures. Studies found that sudden cessation of exercise can increase the risk of having depressive symptoms in healthy people (Lange et al., 2023; Morgan et al., 2018). This may be due to the disrupted tryptophan levels and serotonergic neuronal activation caused by exercise cessation (Morgan et al., 2018). In contrast, physically inactive adolescents adapted to homeschooling life easily during the pandemic and therefore suffered less mental disruption.

Our findings could provide important policy insights into adolescents' mental health. First, school- and family-based health education programs need to be implemented to encourage a healthy lifestyle, such as regular exercise, sufficient sleep, healthy diet, and reduced SST, particularly in the post-pandemic period, to prevent depressive symptoms among adolescents. Second, special attention should be given to adolescents with poor lifestyle behaviors in order to help them develop healthy habits, as they might be vulnerable to depressive symptoms. Third, resources should be allocated to mental health infrastructure (e.g. mental health consultant service at school) to provide adolescents with easily accessible mental health support services, particularly during the potential future pandemic outbreak period, to prevent depressive symptoms. Fourth, given the co-occurrence of related unhealthy lifestyle behaviors, policy-makers should consider integrated approaches to foster multiple healthy behaviors among adolescents rather than using an isolated approach that focuses on a single behavior.

To the best of our knowledge, this is the first comparative study on the associations between lifestyle clusters and depressive symptoms during and after the zero-COVID policy period, using 2-year longitudinal data of a large sample of adolescents in Ningbo, China. However, there are several limitations in our study. First, the lifestyle behaviors of the adolescents were measured by self-reported questionnaires, which might be affected by recall bias and subjectivity. Additionally, the measures of lifestyle behaviors (e.g. MVPA, SST, SSB, and DFF) were assessed using single items rather than multi-item scales. While these measures have been widely used in previous studies (Freije et al., 2021; Lin et al., 2026; Wang et al., 2017), their reliability and validity could not be directly assessed. Future studies may consider using more comprehensive multi-item scales, such as the Physical Activity Questionnaire for Adolescents (PAQ-C) (Moore et al., 2007) and the Food-frequency Questionnaire (FFQ) (Cade et al., 2004), to validate and extend our findings. Second, the present study focused only on adolescents in Ningbo, a coastal city in eastern China, without including participants from other regions, which may affect the generalizability of the findings. Third, while our study compared the associations between lifestyle clusters and depressive symptoms among adolescents in the two separate periods, we did not investigate the transitions of individuals across these clusters over time. To address these limitations, future long-term longitudinal studies may use objective measures (e.g. accelerometers and polysomnography) of lifestyle behaviors to examine lifestyle changes in relation to the depressive symptoms of adolescents based on multiple-center data. Besides, to understand whether and how adolescents transit between healthier and unhealthy clusters would be valuable for developing targeted interventions. Therefore, future research may employ advanced methods such as latent class analysis (LCA) or latent transition analysis (LTA) to explore the dynamic patterns of lifestyle behaviors and their impact on mental health outcomes.

## 5. Conclusion

This comparative study investigated the associations between clusters of lifestyle behaviors and depressive symptoms among Chinese adolescents during and after the zero COVID-19 policy period. We identified five lifestyle clusters, based on adolescents' levels of MVPA, SST, SLD, and SSB and DFF consumption in each period. We found that, in general, unhealthy lifestyle clusters were associated with higher likelihood of depressive symptoms. However, the associations were stronger and more significant in the post-zero-COVID policy period. Our results highlight the importance of interventions in adolescents' lifestyle behaviors to avoid long-term negative effects on their mental health in the post-pandemic period and potential future pandemics.

## Supplementary Material

Supplementary Material Supplementary Material.docx

## Data Availability

The data that support the findings of this study are available upon request from the corresponding authors. The data are not publicly available due to privacy and ethical restrictions.

## Notes

Institutional Review Board Statement: The study was conducted in accordance with the Declaration of Helsinki and was approved by an Institutional Review Board/Ethics Committee. See details under Methods.

The study received an exemption from an Institutional Review Board/Ethics committee; See details under Methods.
