# Supplementary material for: Lifestyle behavior clusters and their associations with depressive symptoms among Chinese adolescents: during and after COVID-19 period
Source: Health Psychol Behav Med. 2026 Jun 1;14(1):2677983. doi: 10.1080/21642850.2026.2677983 (PMC13228188; doi:10.1080/21642850.2026.2677983)
Supplement: Supplementary Material — docx [file RHPB_A_2677983_SM9069.docx]

| **Zero-COVID Policy Period (2022)** | | | | | |
| --- | --- | --- | --- | --- | --- |
|  | **Cluster 1** | **Cluster 2** | **Cluster 3** | **Cluster 4** | **Cluster 5** |
| Lifestyle Behaviors  Cluster Name | Healthy Lifestyle | Physical inactivity | Physical inactivity-Insufficient sleep | Physical inactivity-Excessive SST-Insufficient sleep | Excessive SSB-Physical Inactivity-Insufficient Sleep |
| No beverage | 90.40% | 100.00% | 100.00% | 100.00% | 19.20% |
| Low screen time | 84.20% | 100.00% | 100.00% | 0.00% | 56.20% |
| Regular physical activity | 100.00% | 0.00% | 0.00% | 0.00% | 0.00% |
| Sufficient sleep duration | 50.90% | 100.00% | 0.00% | 48.80% | 35.40% |
| No fried fastfood | 95.20% | 100.00% | 100.00% | 100.00% | 57.90% |
| **Zero-COVID Policy Period (2022)** | | | | | |
|  | **Cluster 1** | **Cluster 2** | **Cluster 3** | **Cluster 4** | **Cluster 5** |
| Lifestyle Behaviors  Cluster Name | Physical inactivity | Insufficient sleep | Physical inactivity-Insufficient sleep | Physical inactivity-Excessive SST-Insufficient sleep | Excessive SSB-Physical Inactivity-Insufficient Sleep |
| No beverage | 100.00% | 91.20% | 100.00% | 100.00% | 16.10% |
| Low screen time | 100.00% | 80.50% | 100.00% | 0.00% | 57.88% |
| Regular physical activity | 0.00% | 100.00% | 0.00% | 0.00% | 0.40% |
| Sufficient sleep duration | 100.00% | 45.40% | 0.00% | 40.00% | 37.40% |
| No fried fastfood | 100.00% | 96.00% | 100.00% | 100.00% | 62.60% |
| **Note:** Percentages denote the probabilities of adolescents from different lifestyle clusters having the listed lifestyle behaviors. | | | | | |

**Supplementary Table 1.** Details on the Lifestyle Clusters during and after the zero-COVID policy period
